# Differences in utilization of Intracytoplasmic sperm injection (ICSI) within human services (HHS) regions and metropolitan megaregions in the U.S.

**DOI:** 10.1186/s12958-017-0263-4

**Published:** 2017-06-12

**Authors:** Pavel Zagadailov, Albert Hsu, David B. Seifer, Judy E. Stern

**Affiliations:** 1 Department of Ob/Gyn, Dartmouth-Hitchcock Medical Center, Lebanon, NH 03756 USA; 2 Department of Ob/Gyn, Dartmouth-Hitchcock Medical Center and the Geisel School of Medicine at Dartmouth, One Medical Center Dr, Lebanon, NH 03756 USA

**Keywords:** ICSI, IVF, Health and human services region, Megaregion, Live birth rate, Utilization

## Abstract

**Background:**

Anecdotal evidence suggests that US practice patterns for ART differ by geographical region. The purpose of this study was to determine whether use of ICSI differs by region and to evaluate whether these rates are correlated with differences in live birth rates.

**Methods:**

Public data for 2012 were obtained from the Centers for Disease Control and Prevention. Clinics with ≥100 fresh, non-donor cycles were grouped by 10 nationally recognized Department of Health & Human Services regions and 11 metropolitan Megaregions and were compared for use of ICSI, frequency of male factor infertility, and live birth rate in women <35 years.

**Results:**

There were 274 clinics in the Health & Human Services regions and 247 in the Megaregions. ICSI utilization rates in Health & Human Services groups ranged between 52.5–78.2% (*P* < 0.0001). Live birth rates per cycle in women <35 years differed (34.1–47.6%; *P* < 0.0001) but did not correlate with rates of ICSI (R^2^ = 0.2096; *P* = 0.18) per cycle. For Megaregions, rates of ICSI per cycle differed (63.4%–93.5%, *P* < 0.0001) as did live birth rates per cycle for women <35 (36.0%–59.0%, *P* = 0.001) but there was only minimal correlation between them (R^2^ = 0.5347; *P* = 0.01). Highest rates of ICSI occurred in Front Range (93.5%) and Gulf Coast (83.1%) Megaregions. Lowest rates occurred in the Northeast (63.4%) and Florida (64.8%) Megaregions. Male factor infertility rates did not differ across regions.

**Conclusions:**

ICSI utilization and live birth rates per cycle for each clinic group were significantly different across geographical regions of the U.S. However, higher ICSI utilization rate was not associated with higher rates of male factor infertility nor were they strongly correlated with higher live birth rates per cycle. Studies are needed to understand factors that may influence ICSI overutilization in the U.S.

## Background

The field of assisted reproductive technologies (ART) continues to grow exponentially after the first successful pregnancy via in-vitro fertilization (IVF) in 1978 which resulted in the live birth of a healthy baby [1]. From 2009 to 2014 the number of ART cycles in the U.S. increased from 146,244 to 208,604 [2]. With the incidence of infertility on the rise [3], use of ART is expected to increase further in the coming years in spite of access limitations. Further, recent innovation and advances in ART methods, ovarian stimulation protocols [4–6], diagnostic procedures [7], and genetic and morphokinetic markers of embryogenesis [8–10] have translated to substantial improvements in clinical outcomes including implantation, pregnancy and live birth rates.

Along with increases in other aspects of ART, the use of ICSI, which was originally developed for severe male factor infertility, has increased substantially over the years and is now often used for indications other than male factor infertility [11–13]. Studies are mixed on whether intracytoplasmic sperm injection (ICSI) is superior to conventional IVF with respect to ART pregnancy and live birth outcomes [14–17]. However, access to ICSI versus conventional IVF varies widely and is dependent upon individual medical insurance policy, federal legislation and state regulations [18–22].

Anecdotal evidence suggests that US practice patterns for use of ICSI in ART differ by geographic region. The goal of this study was to determine whether use of ICSI differs by region and in particular, whether there are differences in usage in urban areas, and to evaluate whether rates of ICSI utilization are correlated with differences in the frequency of male factor infertility, clinical pregnancy, and live birth rates between different regions of the US.

## Methods

This retrospective database analysis was conducted using the National Assisted Reproductive Technology Surveillance System (NASS) from the Centers for Disease Control and Prevention (CDC). Publically available data for 2012 from the NASS were downloaded from the Centers for Disease Control and Prevention (CDC) website [23]. NASS data were grouped according to Department of Health & Human Services (HHS) regions [24] and U.S. Megaregions [25] based on the published address of each clinic and using corresponding ZIP codes. The study received a designation as “not human subjects research’ from the Committee for the Protection of Human Subjects at Dartmouth College.

HHS is represented nationally through 10 regional offices that directly serve multiple states by providing guidance regarding current challenges and actively conduct public health research initiatives. The breakdown is used to address strategic planning for specific national problems over a 4-year period [24]. HHS regions cover 100% of the U.S. population. The Megaregions concept was developed by the Regional Plan Association (RPA). Each Megaregion represents a conglomerate of the cities and metropolitan areas with a common pattern of infrastructural and economic development [25]. Megaregions are specific to densely populated areas and as such the Megaregions cover 77% of the U.S. population.

Clinics with ≥100 fresh, non-donor cycles were included and grouped by region according to the 10 nationally recognized HHS regions and 11 Megaregions. Utilization of ICSI by clinics within each HHS region and Megaregion was correlated with ICSI utilization and with live birth rate per cycle in women <35 years. Usage was compared using ANOVA. Frequency of male factor infertility at each clinic was also evaluated. Regional clinic ICSI rates per cycle were compared with clinic implantation, pregnancy and live birth rates per cycle in women <35 using correlation coefficients. A sensitivity analysis was performed to evaluate whether low numbers of clinics in some of the Megaregions unduly influenced results.

## Results

There were 274 clinics with ≥100 fresh, non-donor cycles within the 10 HHS regions (Table 1). The number of clinics per region ranged from 8 to 55. With respect to Megaregions, there were 247 clinics included with 2 to 79 clinics per region.

**Table 1:** Population, Region and Clinic Numbers in HHS Regions and Megaregions

HHS Regions	US states	Population^a^ (millions)	Clinics (N)
1.Boston	CT, ME, MA, NH, RI, VT	14.6	18
2.New York	NJ, NY	28.4	42
3.Philadelphia	DE, DC, MD, PA, VA, WV	30.2	25
4.Atlanta	AL, FL, GA, KY, MS, NC, SC, TN	62.4	35
5.Chicago	IL, IN, MI, MN, OH, WI	48.1	43
6.Dallas	AR, LA, NM, OK, TX	43.1	28
7.Kansas City	KS, MO, NE	10.8	11
8.Denver	CO, MT, ND, SD, UT, WY	11.2	8
9.San Francisco	AZ, CA, HI, NV	48.7	55
10.Seattle	AK, ID, OR, WA	13.1	9
Total:		310.6	274
Megaregions	Major US Cities		
1. Great Lakes	Buffalo, Cedar Rapids, Chicago, Cincinnati, Cleveland, Columbus, Dayton, Detroit, Erie, Green Bay, Indianapolis, Louisville, Madison, Milwaukee, Pittsburgh, Rochester (NY)	55.5	51
2. Northeast	Atlantic City, Baltimore, Boston, Norfolk, Newark, New York, Philadelphia, Portland (ME), Providence, Richmond, Washington, Wilmington, Worcester	52.3	79
3. Southern California	Anaheim, Bakersfield, Inland Empire (San Bernardino–Riverside), Las Vegas, Long Beach, Los Angeles, San Diego, Tijuana	24.4	28
4. Texas Triangle	Austin, Dallas–Fort Worth, Houston, Oklahoma City, San Antonio, Tulsa	19.7	16
5. Piedmont Atlantic	Atlanta, Birmingham, Charlotte, Greenville, Knoxville, Memphis, Nashville, Piedmont Triad (Greensboro–Winston-Salem), Research Triangle (Raleigh–Durham)	17.6	19
6. Florida	Fort Lauderdale, Jacksonville, Miami, Orlando, Tampa Bay Area, St. Petersburg	17.3	12
7. Northern California	Fresno, Modesto, Oakland, Reno, Sacramento, San Francisco, San Jose	14	16
8. Gulf Coast	Baton Rouge, Houston, Lafayette, New Orleans, Pensacola	13.4	9
9. Cascadia	Portland (OR), Salem, Seattle, Tacoma	12.4	9
10. Arizona Sun Corridor	Mesa, Phoenix, Tucson	5.6	6
11. Front Range	Albuquerque, Cheyenne, Colorado Springs, Denver, Pueblo, Salt Lake City	5.5	2
Total		237.7	247

^a^HHS Regions are for 2012. Megaregions are for 2010

**Fig. 1:**
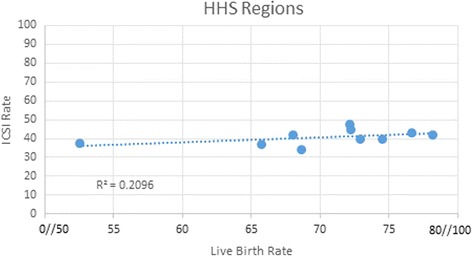
Correlation between ICSI and Live Birth rates in HHS regions in female patients, 35 years old and younger

The mean rate of ICSI per cycle in each clinic group in HHS regions ranged from 52.5 to 78.2% of all clinic cycles (*P* < 0.0001) (Table 2). Implantation rate (range 34.1–44.3, *P* < 0.03) as well as pregnancy (range 39.5–54.2, *P* < 0.03) and live birth rates per cycle (range 34.1–47.6%; *P* < 0.0001) in women <35 years of age also differed. Male factor infertility diagnosis rates per cycle were not significantly different across HHS regions. Correlation coefficients between ICSI and pregnancy (R^2=^0.1467; *P* = 0.27), or live birth (R^2^ = 0.2096:P-0.18) were low. The correlation coefficient for ICSI and implantation rate was higher (R^2=^0.846; *P* = 0.0002). Figure 1 shows the lack of correlation between ICSI and live birth rates.

**Table 2:** IVF Outcomes per HHS Region

HHS Regions	ICSI Rate** (mean % ± SD)	Male Factor (mean % ± SD)ƭ	Implantation Rate* (mean % ± SD)	Pregnancy Rate* (mean % ± SD)	Live Birth Rate** (mean % ± SD)
1.Boston	52.5 ± 19.4	28.8 ± 7.7	34.5 ± 7.6	45.0 ± 8.0	37.5 ± 7.2
2.New York	65.7 ± 20.4	33.8 ± 17.3	35.1 ± 10.7	43.9 ± 9.6	36.9 ± 10.6
3.Philadelphia	68.6 ± 17	32,6 ± 12.5	34.1 ± 9.4	39.5.6 ± 14.0	34.1 ± 11.4
4.Atlanta	72.9 ± 16	38.8 ± 14.6	36.6 ± 6.3	46.2 ± 7.6	40.0 ± 7.6
5.Chicago	74.5 ± 16.3	39.8 ± 15.3	35.5 ± 7.7	45.6 ± 9.1	39.6 ± 9.2
6.Dallas	72.2 ± 18.9	38.5 ± 13.4	40.1 ± 7.1	50.1 ± 9.9	44.8 ± 9.5
7.Kansas City	78.2 ± 15.4	40.0 ± 10.3	39.3 ± 10.0	47.4 ± 8.2	41.8 ± 7.5
8.Denver	72.1 ± 17.1	42.8 ± 12.0	44.3 ± 8.8	54.2 ± 9.5	47.6 ± 8.6
9.San Francisco	76.7 ± 16.5	35.0 ± 18.5	37.9 ± 9.5	49.2 ± 9.8	43.0 ± 9.8
10.Seattle	68 ± 16.6	34,2 ± 12.0	40.9 ± 8.9	46.5 ± 9.9	42.0 ± 9.9

*P* value for columns ƭ =0.12, * < 0.03; ** < 0.0001

**Fig. 2:**
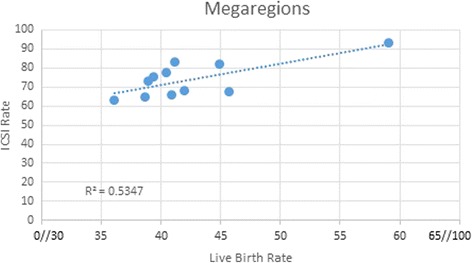
Correlation between ICSI and Live Birth rates in Megaregions in Female patients, 35 years old and younger

In Megaregions, the mean rate of ICSI differed (range 63.4%–93.5%, *P* < 0.0001) as did, implantation rate (range 34.6–55.7, *P* < 0.001), pregnancy rates per cycle (range 42.7–65.5 *P* < 0.001), and live birth rates per cycle (range 36.0%–59.0%, *P* < 0.0001) for women <35 (Table 3) but there was only minimal correlation between them (R^2^ = 0.4349, 0.5687, 0.5347 respectively). Figure 2 shows only a moderate correlation between ICSI and per cycle live birth rates (R2 = 0.5347; *P* = 0.01). Highest rates of ICSI occurred in the Front Range (93.5%) and Gulf Coast (83.1%) Megaregions. Lowest rates occurred in the Northeast (63.4%) and Florida (64.8%) Megaregions. There was no significant difference in male factor infertility rates per cycle across Megaregions.

**Table 3:** IVF Outcomes per Megaregion

Megaregions	ICSI Rate** (mean % ± SD)	Male Factor (mean % ± SD)ƭ	Implantation Rate* (mean % ± SD)	Pregnancy Rate* (mean % ± SD)	Live Birth Rate** (mean % ± SD)
1. Great Lakes	75.3 ± 16.1	38.8 ± 14.6	35.2 ± 7.6	45 ± 8.8	39.3 ± 8.9
2. Northeast	63.4 ± 20.4	31.7 ± 14.4	34.8 ± 9.8	42.7 ± 11	36.0 ± 10.3
3. Southern California	82.3 ± 14.2	30.2 ± 13.3	39.9 ± 9.7	51.6 ± 9.9	44.9 ± 10.5
4. Texas Triangle	67.8 ± 20.3	36.0 ± 10.4	41.7 ± 5.7	50.9 ± 9.2	45.7 ± 8.4
5. Piedmont Atlantic	77.7 ± 12.9	38.5 ± 12.9	37.2 ± 6.4	47.2 ± 9.3	40.5 ± 9.3
6. Florida	64.8 ± 18.6	38.2 ± 17.6	36.2 ± 7.1	45 ± 5.1.0	38.6 ± 5.1
7. Northern California	66.0 ± 16.8	35.9 ± 12.7	35.5 ± 9.6	46.6 ± 8.9	40.9 ± 7.9
8. Gulf Coast	83.1 ± 10.0	34.1 ± 11.7	36.9 ± 9.02	46.3 ± 11.2	41.2 ± 11.2
9. Cascadia	68.0 ± 16.6	34.2 ± 12.0	40.9 ± 8.9	46.5 ± 9.9	42.0 ± 9.9
10. Arizona Sun Corridor	73.0 ± 19.2	38.5 ± 22.9	34.6 ± 8.02	46.6 ± 12.6	39.0 ± 12.4
11. Front Range	93.5 ± 3.5	39.0 ± 19.8	55.7 ± 4.2	65.5 ± 6.2	59.0 ± 1.8

*P* value for columns ƭ =0.20, * < 0.001; ** < 0.0001

## Discussion

This study demonstrates a significant difference in ICSI utilization among both HHS regions and Megaregions (*P* < 0.0001) despite a lack of difference in frequency of the diagnosis of male factor infertility between HHS regions or Megaregions. Additionally, there were significant differences in live birth rates for women under 35 years old (*P* < 0.001), by HHS region and by Megaregion. Nationally, ICSI rates have increased steadily over the past 11 years from 55% in 2003 to 69% in 2014 [11–13]. These recent national ICSI rates are consistent with our study findings. However, it is important to note that our data demonstrate that higher ICSI utilization is not supported by a difference in the frequency of the diagnosis of male factor infertility nor by a corresponding improvement in ART treatment outcomes across HHS and Megaregions. Stated another way, the nearly 50% increase in regional utilization of ICSI does not appear to be based on regional differences in the frequency of male factor infertility. We speculate that ICSI is most likely not beneficial to those without male factor as reflected by the lack of improvement in overall ICSI outcomes.

Use of the HHS regions allowed us to group the clinics such that all regions of the country were represented. By contrast, the Megaregions were used to enable us to evaluate the clinical outcomes in large metropolitan areas. Using both of these regional designations, there were differences in ICSI usage as well as differences in pregnancy and birth outcomes. Although additional designations were available based on insurance coverage, given the complexity of changing state by state and plan by plan coverage we could not determine the proportion of covered cycles in each location. Although Megaregion data included fewer clinics, these were concentrated in metropolitan areas where competition as well as regional interaction may be more concentrated and consistent. This may explain why the differences in Megaregions were more pronounced than those in HHS regions.

Furthermore, although use of ICSI has increased steadily in recent years [11–13], studies vary on whether ICSI is superior to conventional IVF [14–17]. Ming [15] demonstrated a higher fertilization rate for ICSI than for IVF fertilization in randomly assigned oocytes from patients with indications of tubal disease, endometriosis, and ovulatory dysfunction. Komsky-Elbaz et al. [17] found more fertilization failure after IVF than after ICSI, however, they partially stripped the embryos of cumulus cells in both groups which may have reduced the IVF fertilization rate. By contrast, Grimstad et al., [14] using national data from the Society for Assisted Reproductive Technology (SART) found that in tubal ligation patients with no male factor, those using ICSI rather than IVF had lower clinical intrauterine gestation and live birth rates. Butts et al. [16], using SART national data also found no improvement with ICSI. In fact, the use of ICSI was associated with reduced odds of live birth in women with diminished ovarian reserve. The fact that our study showed no association between rates of ICSI and differences in the frequency of male factor as well as only minimal correlation with pregnancy and live birth rates suggests that the use of increased ICSI does not result in increases in success and therefore that ICSI may be overused.

This study has several strengths and limitations. The strength is in the fact that all regions of the country are represented and that over 95% of clinics report to NASS. Limitations include the retrospective nature of the study which does not permit consideration of several variables including patient-specific demographics as well as comorbidities and socioeconomic status. Use of published data from NASS limited our ability to evaluate pregnancy and birth outcomes for all cycles regardless of age since the NASS data publishes clinic specific values by age category. We chose age < 35 as best representing the overall clinical and laboratory success of the clinics studied. Further, the presence of differing insurance coverage within each region and the proportion of private versus hospital-affiliated clinics within a region could not be considered and may be confounding factors. Future research may determine the impact of these variables on ICSI utilization rates and the correlation with live birth rates.

## Conclusion

In summary, these data suggest that ICSI is most likely over-utilized in regions with the greatest utilization in the US. It is speculated that regions with the greatest utilization are regions using ICSI for non-male factor reasons. Furthermore, we speculate that the usage of ICSI does not result in an improvement of outcomes as it is likely employed in cases that do not involve male factor infertility. These data further support the concept that ICSI does not improve outcomes when it is used for non-male factor infertility and supports the contention that it is being over applied. Overutilization of ICSI is associated with increased risk and cost to patients undergoing ART. Further study is needed to identify the reasons which contribute to overutilization of ICSI. Possible explanations may relate to insurance coverage availability, laboratory efficiencies, and/or perceived competition among clinics in specific regions of the country. Another relevant consideration for further examination is the specific criteria used to define male factor infertility and the indication for ICSI used by clinics in different regions of the country.

## Abbreviations

ART: Assisted Reproductive Technology
IVF: In-Vitro Fertilization
ICSI: Intracytoplasmic Sperm Injection
NASS: National Assisted Reproductive Technology Surveillance System
CDC: Centers for Disease Control and Prevention
HHS: Department of Health & Human Services
ANOVA: Analysis of Variance
SART: Society for Assisted Reproductive Technology
